# Does extended follow-up period after birth improve precision of diagnosis of congenital anomalies? An observational study based on the Berlin Embryotox project

**DOI:** 10.1007/s00431-025-06333-z

**Published:** 2025-07-22

**Authors:** Corinna Weber-Schoendorfer, Nadia Maaß, Lukas Lohse, Evelin Beck, Christof Schaefer, Katarina Dathe

**Affiliations:** Charité – Universitätsmedizin Berlin, corporate member of Freie Universität Berlin and Humboldt-Universität zu Berlin, Institute of Clinical Pharmacology and Toxicology, Embryotox Centre of Clinical Teratology and Drug Safety in Pregnancy, Augustenburger Platz 1, 13353 Berlin, Germany

**Keywords:** Congenital abnormalities, Birth defects, Abnormalities, Drug-induced, Pregnancy, Follow-up studies, Delayed diagnosis

## Abstract

The aetiology of congenital anomalies is often difficult to assess. Genetic abnormalities can play a role or exogenous factors such as maternal medication during pregnancy. Observational studies require reliable data on both gestational time and dosage of drug exposure during pregnancy and, if applicable, on precise description of congenital anomalies in the infant. The Berlin Embryotox Centre carried out a questionnaire-based project to investigate whether longer follow-up periods after birth lead to more accurate diagnoses of congenital anomalies. The Berlin Embryotox Centre offers risk assessment on medication during pregnancy to health care providers and pregnant women. Follow-up questionnaires asking for course and outcome of pregnancy are routinely sent out 8 weeks after the estimated date of birth. In this project, three additional questionnaires were sent to women with a singleton live-born infant, asking for paediatric findings documented along routine examinations offered to all children in Germany at the age of approximately 6 months (U5), 1 year (U6) and 2 years (U7). In addition, parents were asked to report their observations on the child’s development. Data were collected between March 2019 and May 2024. A total of 3719 parents completed at least one of the three additional follow-up questionnaires. Results of the standard questionnaire 8 weeks after birth showed 138 infants with a major defect and 13 with a genetic disorder. At the end of the extended follow-up period, 180 children were reported to have a major birth defect and 39 a genetic disorder. The rate of major birth defects increased from 3.7% to 4.8%. Gain of information was largest between the examinations at 8 weeks and 6 months.

*Conclusion*: Extending the observation period beyond the neonatal period substantially improves diagnostic accuracy in terms of completeness and specification of congenital anomalies including genetic disorders. Studies on the risk and safety of drugs in pregnancy would benefit considerably from routine follow-up for at least 6 months after birth.

**Table:** 

**What is Known:** • *Diagnosis of congenital anomalies is often incomplete during the neonatal period*. • *A longer follow-up period increases the completeness of recorded congenital anomalies*.	
**What is New:** • *Regarding major birth defects, gain of information after the neonatal period is largest up to the age of 6 months*. • *Only one-third of genetic diseases were diagnosed during the neonatal period, a further third up to the age of 6 months*.

**Supplementary Information:**

The online version contains supplementary material available at 10.1007/s00431-025-06333-z.

## Introduction

Dependent on the definition of congenital anomalies and the method of data collection, between 1.8% and 6.9% of all infants have major congenital anomalies [1–3]. Congenital anomalies can have a lifelong impact on the affected individual and his/her family. Often, it is difficult to determine why a child is affected. Monogenetic or chromosomal abnormalities, maternal diseases such as poorly controlled diabetes mellitus, intrauterine infections and environmental factors such as pollution or maternal medication, particularly during the first trimester of pregnancy, may play a role [4–6].

Although an increasing portion of malformations is detected by prenatal ultrasound [7, 8] and almost 50% are diagnosed in the first four weeks of life, around a third are not detected until after the neonatal period [9, 10]. This may be due to unfavourable diagnostic conditions in the neonatal period, e.g. because the infant cries during the physical examination. Even oral clefts are not always diagnosed at birth [11]. Cardiovascular, urogenital tract and other internal structural anomalies may remain undetected during the neonatal period because infants do not routinely receive an echocardiogram and an ultrasound of the abdomen.

For more than 30 years, the Berlin Embryotox Centre of Clinical Teratology and Drug Safety in Pregnancy (Embryotox) has been collecting data on the course and outcome of drug-exposed pregnancies as a basis for observational studies on the effect of drugs on the unborn child [12]. The rate of major birth defects is a main focus of these studies [13–15]. The Berlin Embryotox Centre regularly collects information on the child’s development up to the third paediatric check-up at 4 to 5 weeks (U3 examination).

The intention of this pilot project is to analyse whether extending the follow-up period after birth leads to more accurate information on major birth defects and if implementation of longer periods in routine follow-up appears reasonable. Results of genetic tests are often only available far beyond the neonatal period. Therefore, longer follow-up may contribute to the information on specificity and severity of birth defects and anomalies and facilitate differentiation between genetic and environmental causes.

To evaluate the information gain of extended follow-up periods, the Berlin Embryotox Centre conducted a questionnaire-based study.

## Methods

The Berlin Embryotox Centre serves as a national clearing centre for drug risks in pregnancy. It is publicly funded and offers free advice to pregnant women and health care professionals. Therefore, a person in need of advice will make contact with the Embryotox Centre on their own initiative. Using a structured approach during the counselling process, relevant patient data are collected, including maternal characteristics, drug exposures, treatment indications, obstetric and family history. Thus, the Embryotox cohort comprises cases generated through physicians or patients approaching the centre in regard to their requests concerning risk and safety information during pregnancy (‘passive case finding’).

### Embryotox routine follow-up-process

With the patient’s consent, follow-up on further course and outcome of pregnancy is initiated at first contact, mainly in pregnancies exposed to medication in the first trimester. Follow-up data are prospectively ascertained, i.e. neither the outcome of pregnancy nor prenatal pathology is known at follow-up initiation. Standardised questionnaires are sent out 8 weeks after the expected date of birth (EDOB). At this time, the third paediatric check-up (U3) at 4 to 5 weeks after birth has taken place. Almost all infants in Germany are covered by this examination.

The follow-up questionnaire includes information on maternal drug use, complications during pregnancy, gestational age at birth, neonatal outcome, sex, birth weight, head circumference, umbilical cord artery pH, Apgar scores and details of congenital anomalies and postnatal conditions and, if applicable, details of pregnancy loss (miscarriages, stillbirths, terminations of pregnancy). In cases of missing or implausible data, further medical records are requested. All data are archived in the Embryotox database (VigilanceOne PharmApp Solutions GmbH). Approximately 3000 follow-up procedures are completed every year.

### Additional 2-year follow-up

In this project, we focused on pregnancies with a singleton live-born child and complete follow-up after 8 weeks. From March 2019 until August 2023, we sent an invitation (‘starter pack’) to mothers of singleton live-born children with a postal address in Germany. Cases were retrieved from the Embryotox database at the infant’s age of 7 months. The starter pack included information about the project, a form on data protection and consent and a postage-paid return envelope. A valid e-mail address had to be provided along with the consent form. Once formal consent had been obtained, the process took place digitally: Parents received a link to a personalised questionnaire via e-mail. Timing and content of the questionnaires considered the time schedule of the paediatric check-ups offered to all children in Germany (‘U-examinations’). Paediatricians document their findings in a booklet (‘Your child’s medical records’), edited by the Federal Joint Committee [16].

The respective follow-up links were sent to the participants at the time of the U5 examination (at the age of 6th to 7th month), U6 examination (10th to 12th month) and the U7 examination (21st to 24th month). All data received by 21 May 2024 were included in the analysis.

The first part of the respective questionnaire asked for the infant’s growth parameter and the paediatrician’s assessment. The structure and items of this part largely followed that of the examination booklet; for details, see the link to the English translation of the examination booklet in the References section [16]. Parents were asked to copy the paediatrician’s entries from the booklet. The second part of the questionnaire asked for the parent’s view of all relevant congenital anomalies or postnatal diseases of the child since birth, the date of diagnosis, the course of the disease and, if applicable, therapeutic measures.

The REDCap web application (https://projectredcap.org/) was used to manage and store the online questionnaires completed by the parents.

### Review of returned information and classification of congenital anomalies

All relevant information on congenital malformations was extracted from the returned questionnaires. If the data was implausible or inaccurate, the study team contacted the parents again to obtain further medical information and documents.

Reported anomalies were categorised as major birth defects, minor anomalies or genetic diseases by two experts of the project team following the EUROCAT guidelines [17]. For each enrolled child, all returned questionnaires U3 up to U7 were considered.

Children with a genetic disorder and a major birth defect that is not related to the genetic disorder, such as ventricular septal defect (VSD) and cystic fibrosis, were categorised as major birth defects and genetic disorders. Major birth defects interpreted as part of a genetic syndrome, however, were assigned to genetic disorders only.

### Statistical analysis

Birth defect rates were calculated by dividing all observed major birth defects among live births by the number of all live births.

Descriptive analyses were performed. Numerous parameters were evaluated to describe the maternal characteristics of the study cohort. Median/mean and IQR were calculated for maternal age, BMI and gestational week (GW) at first contact with the Embryotox Centre. Absolute and relative frequencies in percentage (%) were calculated for maternal educational achievement, alcohol and nicotine consumption, number of previous pregnancies and previous miscarriages and previous children with malformations. Similar analyses were performed for neonatal characteristics such as gestational age at birth, infant’s head circumference and weight. Absolute and relative frequencies in percentage (%) were given for the data on sex and prematurity.

**Fig. 1:**
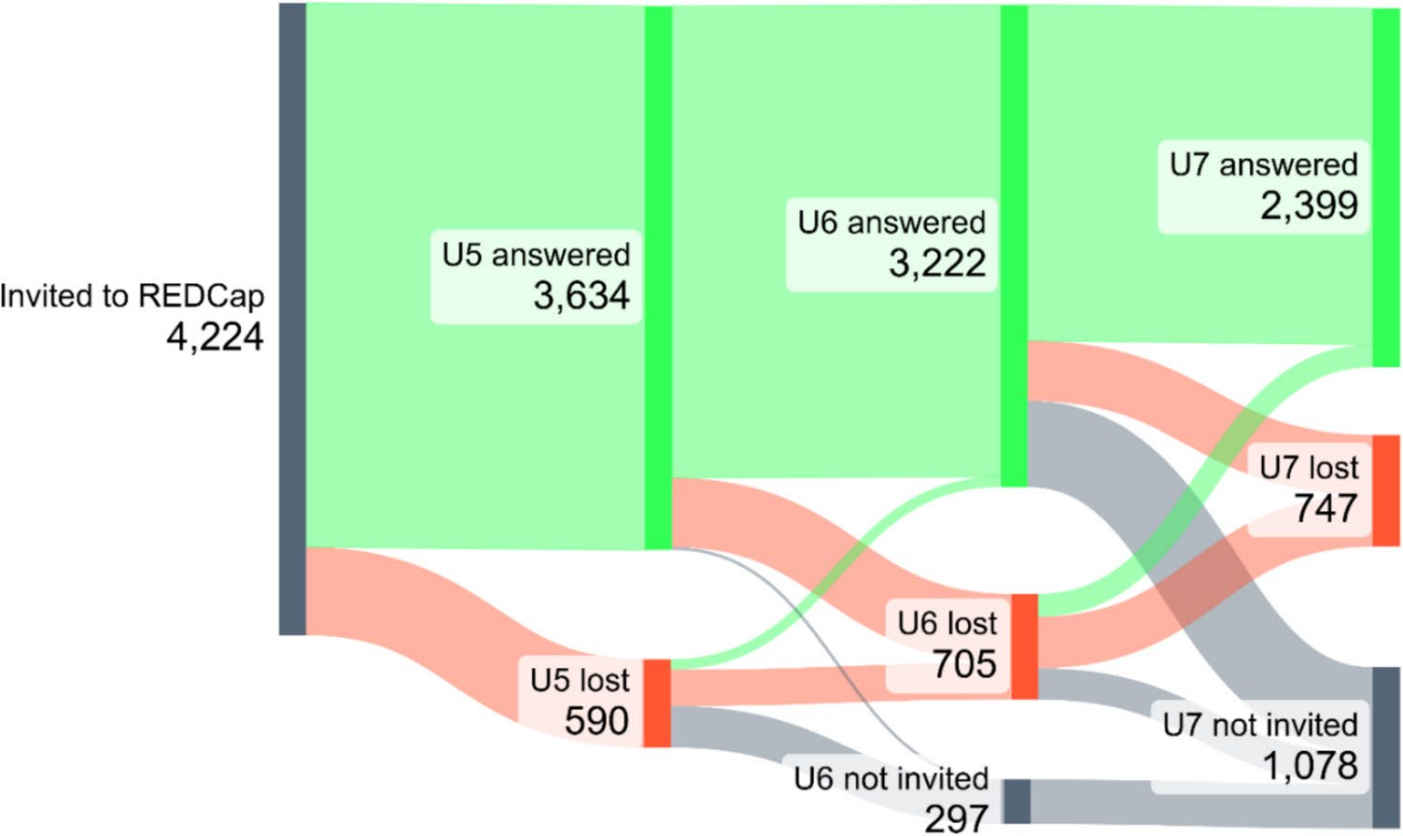
Number of completed questionnaires at the three timepoints (U5, U6, U7)

Standard deviation scores (SDS) for comparison of biometrical data by infant major birth defect status were calculated using the LMS method, i.e. assuming the data to be normally distributed with parameters M, S after being Box-Cox transformed with parameter L. The LMS method is described in detail in [18]. Reference parameter values were taken from the KiGGS study (German Health Interview and Examination Survey for Children and Adolescents) [19] linearly interpolated with a least squares approximation of LMS values for the birth percentiles in Voigt et al. [20]. This allows comparison of children’s weight and head circumference at different ages and across sex. Figure 1 was created with SankeyMATIC (https://sankeymatic.com/about/). Other plots and calculations were performed with R 4.3.2 [21].

## Results

### Response rate of questionnaires

Of the 9199 invitations (starter packs) to join the study, 4224 gave their consent to participate in the study. Four hundred thirty-four invitations did not reach the addressee. Three thousand seven hundred nineteen of the 4224 completed at least one questionnaire, and 505 did not participate. Thus, 48% (4224 of 8765) agreed to participate, and 42% (3719 of 8765) completed at least one of the questionnaires.

In total, there were 3634 completed U5 questionnaires (6th to 7th month of life), 3222 U6 questionnaires (10th to 12th month) and 2399 U7 questionnaires (21st to 24th month). The response rate was 86% (3634 of 4224 invited to participate) for the U5 questionnaire, 82% (3222 of 3927) for the U6 questionnaire and 76% (2399 of 3146) for the U7 questionnaire. Dynamics of returned questionnaires is shown in Fig. 1.

### Maternal characteristics of the project cohort

Maternal characteristics at the time of first contact were compared between those who did not respond (*n* = 4541), those who completed at least one questionnaire (*n* = 3719) and those who gave consent but did not participate (*n* = 505). The observed differences between the three groups were minor. Participants were more often first-time parents and contacted Embryotox slightly later in pregnancy (GW 12.4 vs. GW 10.3). Fewer of them smoked, and they tended to have a higher level of education than those who did not respond (Table S1).

### Neonatal characteristics

There were no significant differences in infant characteristics at birth between the three groups defined above (Table S2).

In order to validate the representativeness of our study cohort, we also compared relevant characteristics with the German population statistics [22]. The ratio of male-to-female newborns differed only slightly (male: 50.5% vs. 51.3%; female 49.5% vs. 48.7%), and the preterm birth rate was similar (6.4% vs. 6.2%). There were slightly fewer small for gestational age (SGA) infants in the study cohort (8% vs. 9.3%) (Table S3). Birth weights of the 3719 children in the study cohort were similar to those reported in German population statistics [20] (Fig. S1).

### Rate of major birth defects over a period of 2 years

We evaluated the reported anomalies and birth defects case by case over time during the follow-up period. Birth defects may later turn out to be misdiagnosed, less or more severe than initially assumed (major birth defect versus minor anomaly) or resolve such as a spontaneously closed atrial septal defect (ASD). Finally, birth defects may turn out to be a symptom of a genetic disease and reassigned from major birth defect to the group of genetic anomalies.

At the U3 examination, there were 138 major birth defects among the 3719 responders (3.7%). At the U5, 32 additional major birth defects were reported, a further 14 at the U6 examination and another four at the U7 examination.

Of the total of 188 children with a major malformation, there were six ASD that spontaneously closed within the first 6 months after birth. According to EUROCAT, they were not classified as ‘major’. Furthermore, two children diagnosed with major birth defects at U3 and U5 later turned out to be affected by a genetic disorder: one child with DiGeorge syndrome and one with autosomal dominant polycystic kidney disease (ADPKD).

Up to the U7, the major malformation rate rose to 4.8% (180/3719). The increase of reported major malformations was highest from the U3 (4 to 6 weeks) to the U5 (6th to 7th month): 138/3719 to 164/3719. Table 1 gives an overview of the rates of major birth defects by U-examination during the study period and their assignment to organ subgroups or subcategories.

**Table 1:** Cumulative numbers of reported major birth defects during the follow-up period by EUROCAT organ system subgroups

Examinations	U3	U5	U6	U7
Total (*n*), infants affected by major birth defects	138/3719^a^	164/3719^a^	177/3719^a^	180/3719^a^
Organ system/category				
Nervous system anomalies	5	6	7	7
Eye anomalies	2	3	4	4
Ear, face and neck anomalies	1	3	3	4
Congenital heart defects	66	74^b^	79	81
Respiratory anomalies	0	0	0	0
Oro-facial clefts	3	3	3	3
Gastro-intestinal anomalies	6	7	7	7
Abdominal wall defects	0	0	0	0
Congenital anomalies of kidney and urinary tract	31	38	42	42
Genital anomalies	16	18	19	20
Limb anomalies	16	18	18	18
Other anomalies/syndromes	3	6	7	7
Total (*n*), per affected organ system/category	149	176	189	193

Legend. Multiple major malformations in one infant affecting different subgroups are counted separately

^a^Denominator of U3 examination was kept throughout follow-up period although the number of responders decreased from U3 to U7; see the ‘Discussion’ section

^b^ASD II is only considered a major malformation ‘if the flow across the defect is still present at 6 months after birth, corrected for gestational age in preterm infants’ [17]. Six of the ASD II closed spontaneously, but 14 other infants with heart defects were additionally reported (66–6 + 14 = 74)

### Genetic disorders

A total of 39 children were reported with a genetic disorder; most of them were diagnosed after the U3 examination. Only 13 were identified before or at the U3 check-up. Two of them, one infant with spherocytic anaemia and one with cystic fibrosis, had additional unrelated major anomalies and were therefore also assigned to this group. By U5, another 12 children had been diagnosed with genetic disorders. One child with confirmed autosomal recessive sensorineural hearing loss had a double kidney classified as a major birth defect. At the U6 check-up, nine additional children and, at the U7 examination, five children were diagnosed with genetic disorders. For further details, we refer to Table 2.

**Table 2:** List of reported genetic disorders and time of diagnosis

Genetic disorders	Reported at
Cystic fibrosis	U3
Deficiency of methylenetetrahydrofolate reductase (MTHFR) activity	U3
Glucose-6-phosphate dehydrogenase deficiency	U3
Hereditary spherocytosis	U3
Ichthyosis, familial^a^	U3
Ichthyosis, autosomal recessive (ARCI)	U3
Medium chain acyl-CoA dehydrogenase deficiency (MCADD)	U3
Prader-Willi syndrome	U3
Sensorineural familial congenital hearing loss	U3
Trisomy 13	U3
Trisomy 18	U3
Trisomy 21	U3
Very long chain acyl-CoA dehydrogenase deficiency (VLCADD)	U3
Blepharophimosis-ptosis-epicanthus inversus syndrome	U5
Cystic fibrosis	U5
Ehlers-Danlos syndrome	U5
Ichthyosis^a^	U5
Malignant hyperthermia	U5
Maturity Onset Diabetes of the Young (MODY diabetes)	U5
Neurofibromatosis, type 1 (two infants)	U5
Sensorineural familial congenital hearing loss	U5
Short stature, familial^a^	U5
Thalassaemia, alpha and beta minor	U5
Vanishing-testes syndrome^a^	U5
Autosomal dominant polycystic kidney disease (ADPKD)	U6
Beckwith-Wiedemann syndrome	U6
Benign familial haematuria	U6
Dravet syndrome	U6
Familial hypocalciuric hypercalcaemia	U6
Megalencephalic leukoencephalopathy with subcortical cysts	U6
Muckle-Wells syndrome	U6
Noonan syndrome	U6
Short stature, familial^a^	U6
Dentinogenesis imperfecta	U7
DiGeorge’s syndrome	U7
Duane Retraction syndrome	U7
Muscular dystrophy, type Duchenne	U7
Trisomy 9 Mosaic	U7

Legend. Thirteen diagnoses were already known at the U3 examination; 12 were additionally known at the U5 examination; 9 at the U6 and 5 at the U7 examination

^a^Clinical diagnosis

### Comparison of infant biometric data within the study cohort

Head circumferences and body weights of the children at birth and at the U5, U6 and U7 examinations were compared between those with major birth defects and those neither affected with birth defects nor genetic disorders. The weight-SDS of children with and without major birth defects or genetic disorders was very similar at birth. However, the U5 to U7 examinations showed some differences. Children with major birth defects were on average around 0.2 SDS smaller (Fig. S2 and Table S4). For a 6-month-old boy, this would roughly correspond to a 190 g lower body weight and 280 g less at the age of 2 years. No such trend could be observed for head circumferences (Table S5).

## Discussion

The extension of the follow-up observation period from 8 weeks after birth to 24 months has confirmed the expectations placed in the project. The cumulative number of major birth defects detected in the study cohort (*n* = 3719) increased from 138 to 180. The rate of reported major malformations increased from 3.7% to 4.8%.

### Non-participation bias

The 42% participation rate in this study is comparable to that of the Dutch ‘PRegnancy and Infant DEvelopment’ (PRIDE) study (47%) during their pilot period [23] and the Norwegian Mother and Child Cohort Study (MoBa) (41%) [24]. A study based on the ‘Danish National Birth Cohort’ investigated whether a low participation rate (30%) is a source of bias and found no such bias in three selected exposure-risk associations [25].

There were no major differences in maternal characteristics at the time of first contact during pregnancy or in infant characteristics at birth between the three groups: (i) mothers who did not respond (*n* = 4541), (ii) those who completed at least one questionnaire (*n* = 3719) and (iii) those who consented but did not participate (*n* = 505) (Table S1 and S2). Mother–child pairs in this study appear representative for Embryotox users in general. They also appear to be comparable with the total population of pregnant women and their children in Germany (Table S3 and Fig. S1). One exception is that participating mothers were slightly older compared to pregnant women in Germany (33.4 years vs. 31.7 years; Table S3) and achieved a higher level of education (academic degree 58.1% vs. 47.2%, Table S1). This trend, which was already observed in the entire cohort of women contacting Embryotox [26], was more prominent in this longer follow-up project. Other similar research projects such as the PRIDE study mentioned above, a long-term prospective survey to analyse various prenatal and early childhood factors and their influence on the health of mother and child, observed similar characteristics, e.g. 68%–71% of participants had a higher level of education [27, 28].

Participation in follow-up versus non-participation has also been investigated as potential bias in studies on pregnancy outcome after prenatal drug exposure. Neither non-participation nor self-selection of study subjects had a significant impact in other studies on pregnancy outcome after prenatal drug exposure [29, 30].

### Major birth defects

Major birth defect rates observed in this study increased with the age of the children from 3.7% at 1 month after birth (U3) to 4.8% at 24 months (U7). With respect to genetic disorders, only one-third of the 39 children were diagnosed at the U3 examination at the end of the neonatal period.

The denominator determined at the U3 examination was maintained throughout the follow-up period, although the number of responders decreased from U3 to U7 (see Fig. 1). This may result in an underestimation of the major birth defect rate. However, counting only the actual responders as the denominator may result in an overestimation of the birth defect rate. According to our experience, parents of infants with birth defects are more motivated to respond than those with healthy infants. Since an excess of birth defects among non-responders is therefore unlikely, we decided for the former, more cautious approach. Our rate of 4.8% for major birth defects at 24 months is considerably higher than those published by EUROCAT. EUROCAT calculated a prevalence of 1.81% for major birth defects excluding genetic anomalies for live births and stillbirths for the years 2005 to 2022 [1]. The major birth defect rate for live births obtained by the Malformation Monitoring Centre Saxony-Anhalt, Germany, however, was higher with 3.57% in 2023 and 3.88% (95% CI 3.8%–4.0%) between 2011 and 2022 [31].

In our study, the increase of major birth defects with the extension of postnatal follow-up is based on newly reported diagnoses and reassignment of anomalies that were initially classified as ‘minor’. On the other hand, there were spontaneous recoveries (such as closure of ASD) or inaccurate diagnoses of major birth defects that were later removed from this category. Since we used the same criteria from U3 to U7 check-ups, the increase in the malformation rate cannot be explained by a change in classification criteria.

Our study results are in line with other projects. Nevertheless, as the data sources, data presentation and analysis differ, the prevalence rates across monitoring projects should be compared with caution. The results from our long-term surveillance are very close to those from a German active monitoring shortly after birth, with malformation rates including anomalies in pregnancy losses of up to 6.9% [2]. The Massachusetts Birth Defects Monitoring Program, an active malformation surveillance programme, found a malformation rate of 5.1% at birth and of 5.4% at 1 year of age in 2000 [3]. A study based on nationwide Danish health registry data calculated a major birth defect rate of 5.3% at the age of 5 years. At birth, 1.6% of these livebirths were diagnosed with a major malformation, 3.2% up to the age of 3 months and 4.2% at the age of 1 year [32]. A population-based registry study in Sweden investigated the cumulative detection of birth defects in singleton live-born children at various time points from birth to 3 years of age. 1.9% of major malformations were recorded at birth, 3.1% at 90 days, 3.9% at 1 year of age, 4.4% at 2 years and 4.7% at 3 years [33]. A further study analysing data from an Australian Birth Defects Registry showed a cumulative prevalence of major birth defects of 5.1% by 1 year of age and 5.8% by age 6 years. Their results show that 18.7% of all birth defects were diagnosed by ultrasound already during pregnancy; the majority of 47.8% were diagnosed by 1 month of age. Another 20.4% were diagnosed from 1 month up to 1 year and 12.1% between 1 and 6 years of age [9]. Analyses from the South Australian Birth Defects Registry have also demonstrated the benefits of an extended collection period. A total of 81.5% of major defects were diagnosed by 1 year of age, with an additional 17.2% diagnosed between 1 and 5 years of age [10]. The importance of continued follow-up is also supported by the results based on data from the Brigham and Women’s Hospital Surveillance Program. They showed that follow-up for up to 1 year of age improves the quality in the surveillance process. Shortly after birth, major anomalies were initially missed in 3.5% of affected infants, and an additional 2.3% of infants had major malformations that were not detected until later during the first year of life [3].

For teratogenicity studies, all major birth defects should be counted irrespective of their spontaneous or surgical improvement. Otherwise, the drug’s toxicity would be underestimated. Whereas in studies on the health impact of prenatal drug exposure, the final outcome may be more relevant including spontaneous or therapeutic improvement of congenital anomalies. It is of utmost importance that studies comparing exposed vs. non-exposed pregnancies clearly define the duration of follow-up and the inclusion/exclusion of cured/resolved malformations within and across cohorts.

### Strengths and limitations

The Embryotox population could carry a higher birth defect risk due to a selection of high-risk drug-exposed pregnancies. On the other hand, an Embryotox study comparing average drug-exposed pregnancies with non-teratogenic exposures failed to demonstrate a significant risk increase [34]. Furthermore, we cannot rule out a detection bias: Women with a higher level of education, over-represented among Embryotox users, may be more likely to request in-depth investigation of their infants including echocardiography and abdominal ultrasound.

The focus of the paediatric routine examinations is on the age-appropriate development of the child. Once a congenital anomaly has been diagnosed, it is not necessarily mentioned in subsequent U-examinations, whereas the parents were asked to list all anomalies and comment on their course on each questionnaire. The combination of paediatric documents from routine examinations and information provided by the infant’s parents complements each other. In cases of discordance, we had the opportunity to contact the parents and ask for additional (paediatric) information.

## Conclusion

Longer observation intervals beyond the neonatal period significantly improve the diagnostic accuracy in terms of completeness and specificity of congenital anomalies including genetic disorders. Studies on the risk and safety of medication in pregnancy would greatly benefit from additional routine follow-up of at least 6 months after birth.

## Supplementary Information

Below is the link to the electronic supplementary material.

Supplementary file1 (PDF 399 KB)

## Data Availability

The data that support the findings of this study are not openly available due to reasons of sensitivity and are available from the corresponding author upon reasonable request. Data are located in limited access drive at the Charité central data storage.

## Abbreviations

ASD: Atrial septal defect
CI: Confidence interval
GW: Gestational week
EDOB: Expected date of birth
EUROCAT: European network of population-based registries for the epidemiological surveillance of congenital anomalies
SDS: Standard deviation score
SGA: Small for gestational age
U-examination: Paediatric check-ups recommended by the Federal Joint Committee in Germany and documented in ‘Your child’s medical records’ booklet
U3: Paediatric examination at 4th–5th week
U5: Paediatric examination at 6th–7th month
U6: Paediatric examination at 10th–12th month
U7: Paediatric examination at 21st–24th month
VSD: Ventricular septal defect
