# Supplementary material for: Does extended follow-up period after birth improve precision of diagnosis of congenital anomalies? An observational study based on the Berlin Embryotox project
Source: Eur J Pediatr. 2025 Jul 22;184(8):495. doi: 10.1007/s00431-025-06333-z (PMC12283439; doi:10.1007/s00431-025-06333-z)
Supplement: Supplementary file 1 — Supplementary file1 (PDF 399 KB) [file 431_2025_6333_MOESM1_ESM.pdf]

Supplementary file

Does extended follow-up period after birth improve precision of diagnosis of congenital anomalies? An observational study based on the Berlin Embryotox Project

Abbreviations:

- BD, birth defect
- BMI, body mass index
- GW, gestational week
- IQR, interquartile range
- KiGGS, Studie für die Gesundheit der Kinder und Jugendlichen in Deutschland (engl. German Health Interview and Examination Survey for Children and Adolescents)
- SDS, standard deviation score
- U3, paediatric examination at 4th - 5th week
- U5, paediatric examination at 6th - 7th month
- U6, paediatric examination at 10th - 12th month
- U7, paediatric examination at 21st- 24th month

Content

Table S1. Comparison of maternal characteristics 3
Table S2. Comparison of infants' characteristics at birth 4
Table S3. Comparison of selected characteristics between participants and population-based data.... 5
Figure S1. Weight percentiles of participating children compared to population-based data 5
Figure S2. Comparison of birth weight by infant major birth defect status 6
Table S4. Standard deviation scores of weights according to U examination and birth defect status ... 6
Table S5. Standard deviation scores of head circumference according to U examination and birth defect status 7
References 7

Table S1. Comparison of maternal characteristics

N	Participants<br>3,719	No consent<br>4,541	Consent, but no participation<br>505
**Maternal age**, n	3,719	4,540	505
Median age in years (IQR)	33 (31-36)	33 (30-36)	33 (30-37)
**BMI**, n	3,711	4,515	502
BMI (kg/m²) (IQR)	23.2 (21-26.8)	23.3 (21-26.9)	22.8 (20.6-25.8)
**Educational level**, n	2,582	3,581	383
No leaving exam (%)	4 (0.2)	5 (0.1)	1 (0.3)
9 years exam (%)	19 (0.7)	116 (3.2)	4 (1)
10/11 years exam (%)	438 (17)	868 (24.2)	61 (15.9)
Secondary school exam (%)	620 (24)	901 (25.2)	78 (20.4)
Academic study (%)	1,501 (58.1)	1,691 (47.2)	239 (62.4)
**Smoking**, n	3,718	4,539	505
No (%)	3,537 (95.1)	4,163 (91.7)	471 (93.3)
<= 5 cig/d (%)	70 (1.9)	119 (2.6)	11 (2.2)
> 5 cig/d (%)	111 (3)	257 (5.7)	23 (4.6)
**Alcohol**, n	3,716	4,539	505
No (%)	3,474 (93.5)	4,285 (94.4)	469 (92.9)
<= 1 drink/d (%)	120 (3.2)	142 (3.1)	18 (3.6)
> 1 drink/d (%)	122 (3.3)	112 (2.5)	18 (3.6)
**Previous pregnancies**			
**- Previous deliveries**, n	3,719	4,527	505
0 (%)	2,086 (56.1)	2,367 (52.3)	256 (50.7)
1 (%)	1,275 (34.3)	1,594 (35.2)	186 (36.8)
2 (%)	299 (8)	442 (9.8)	45 (8.9)
3 or more (%)	59 (1.6)	124 (2.7)	18 (3.6)
**- Previous miscarriages**, n	3,718	4,523	505
0 (%)	2,870 (77.2)	3,531 (78.1)	384 (76)
1 (%)	598 (16.1)	722 (16)	85 (16.8)
2 or more (%)	250 (6.7)	270 (6)	36 (7.1)
**- Infants with malformations**, n	3,718	4,522	505
0 (%)	3,651 (98.2)	4,439 (98.2)	491 (97.2)
1 (%)	59 (1.6)	79 (1.7)	13 (2.6)
2 or more (%)	8 (0.2)	4 (0.1)	1 (0.2)
**GW at first contact**, n	3,716	4,537	505
**GW at first contact in weeks,** median (IQR)	12.4 (7.6-22.4)	10.3 (6.7-18.7)	11.9 (7.3-20.9)

Legend. BMI, body mass index; d, day; GW, gestational week; IQR, interquartile range.

Table S2. Comparison of infants' characteristics at birth

	Participants	No consent	Consent, but no participation
N	**3,719**	**4,541**	**505**
**GW at birth**, n	3,719	4,541	505
Median GW at birth	39.57	39.43	39.57
**Preterm,** n	3,719	4,541	505
Preterm, n (%)	239 (6.4)	301 (6.6)	28 (5.5)
**Sex,** n	3,719	4,540	505
Female, n (%)	1,841 (49.5)	2,229 (49.1)	260 (51.5)
Male, n (%)	1,878 (50.5)	2,311 (50.9)	245 (48.5)
**Birthweight,** n	3,719	4,541	505
Median weight (IQR)	3,410 (3,110-3,715)	3,400 (3,080-3,735)	3,400 (3,110-3,735)
**Head circumference**, n	3,636	4,270	487
Median head circumference (IQR)	35 (34-36)	35 (34-36)	35 (34-36)

Legend. GW, gestational week; IQR, interquartile range.

Table S3. Comparison of selected characteristics between participants and population-based data

	**Participants**	**Mothers and children in Germany**
Maternal age (mean age in years)	33.4	31.7*
Live-born infants<br>- Male (%)<br>- Female (%)	 50.5<br>49.5	 51.3<br>48.7
Preterm (%)	6.4	6.2
Small for Gestational Age (SGA) (%)	8	9.3
Large for Gestational Age (LGA) (%)	10.3	10

Legend. *Related to all mothers who delivered a child in Germany in 2022. [1] (Table 12612-0015)

Figure S1. Weight percentiles of participating children compared to population-based data

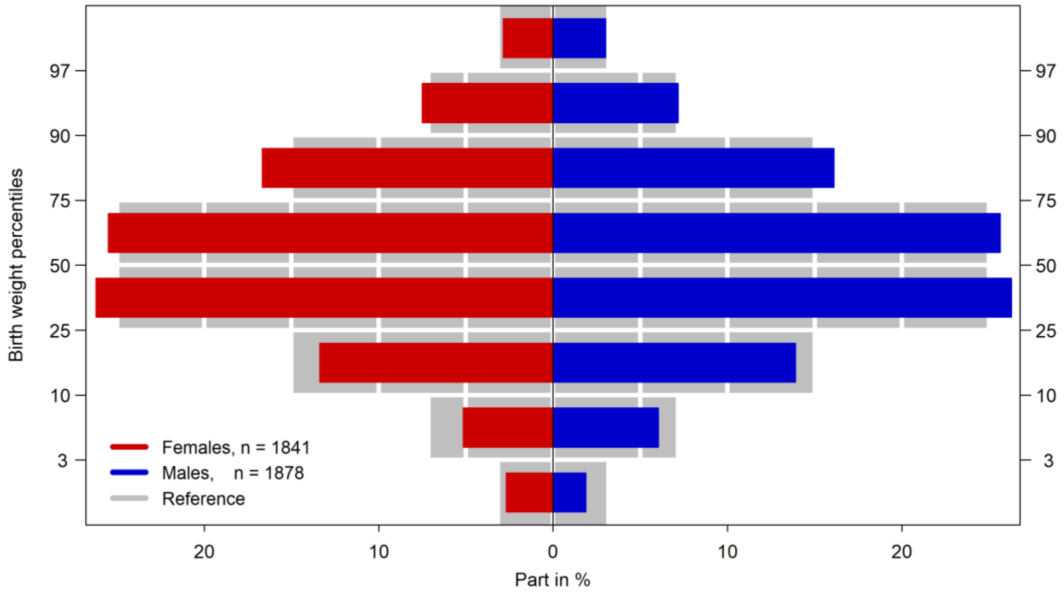

Legend. A comparison of the birth weight percentiles of the 3,719 children in this project with data from Germany (displayed as a gray bar in the background) [2].

Figure S2. Comparison of birth weight by infant major birth defect status

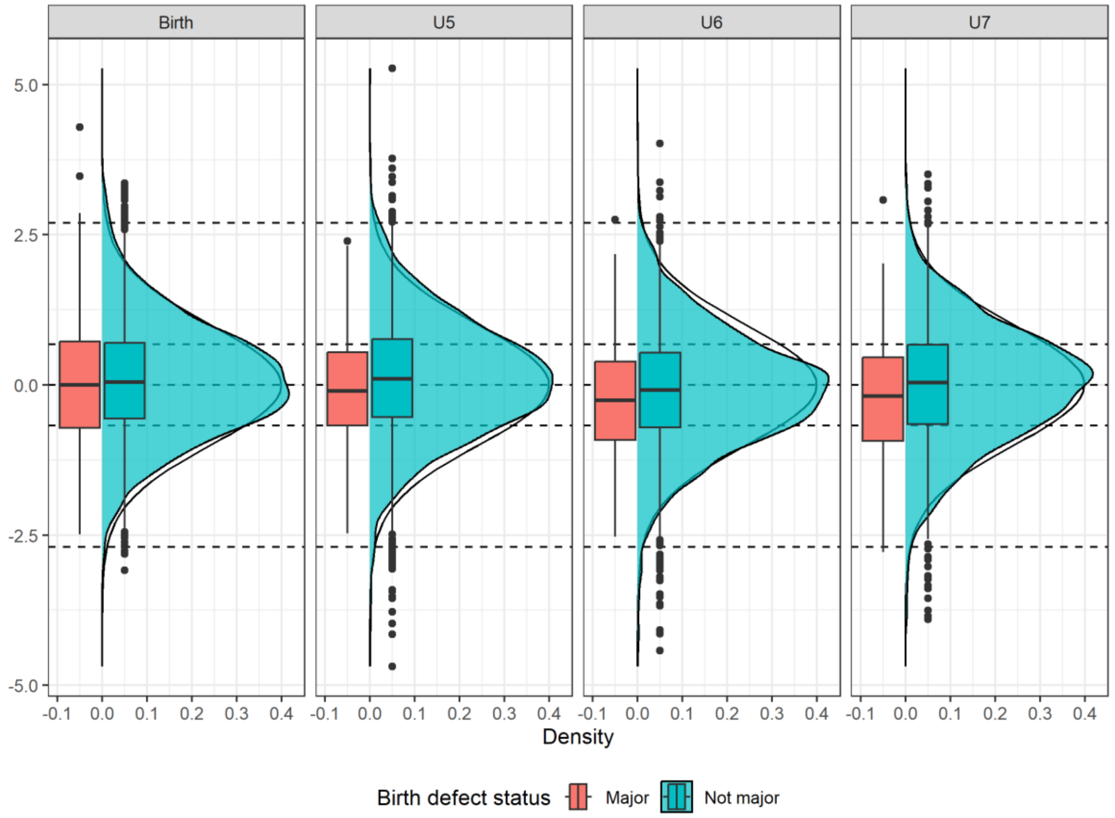

Legend. Standard deviation score (SDS) of weights for children without genetic disorders at each U-examination divided by major birth defect status. For children without a birth defect, representation of a density estimator. In the background, the standard normal distribution representing the German reference cohorts of KiGGS (*engl.* German Health Interview and Examination Survey for Children and Adolescents) [3] and dotted references for the boxplots, also with respect to the standard normal distribution.

Table S4. Standard deviation scores of weights according to U examination and birth defect status

	Birth defect status	n	mean	median	sd
At birth	*Major BD no*	3503	0.084	0.049	0.967
At birth	*Major BD yes*	177	0.072	0.000	1.125
U5	*Major BD no*	3345	0.117	0.103	1.017
U5	*Major BD yes*	171	-0.060	-0.103	1.020
U6	*Major BD no*	2937	-0.085	-0.083	1.005
U6	*Major BD yes*	141	-0.240	-0.254	1.051
U7	*Major BD no*	2121	-0.010	0.038	1.017
U7	*Major BD yes*	104	-0.221	-0.185	1.088

Legend. BD, birth defect. The median is more robust against outliers and should potentially be preferred for this data.

Table S5. Standard deviation scores of head circumference according to U examination and birth defect status

	Birth defect status	n	mean	median	sd
At birth	*Major BD no*	3423	-0.060	-0.077	0.947
At birth	*Major BD yes*	174	-0.045	-0.077	1.076
U5	*Major BD no*	3320	0.159	0.194	1.050
U5	*Major BD yes*	170	0.063	0.115	1.036
U6	*Major BD no*	2895	0.080	0.121	1.064
U6	*Major BD yes*	138	0.019	0.036	1.131
U7	*Major BD no*	2026	0.067	0.089	1.033
U7	*Major BD yes*	102	0.186	0.153	1.190

Legend. BD, birth defect. The median is more robust against outliers and should potentially be preferred for this data.

References

1. Statistik der Geburten (2024). 12612-0001 Lebendgeborene: Deutschland, Jahre, Geschlecht. Statistisches Bundesamt (destatis), Wiesbaden. https://www-genesis.destatis.de/genesis/online?operation=statistic&levelindex=0&levelid=1719478091247&code=12612#abreadcrumb.
2. Voigt M, Rochow N, Schneider KT, Hagenah HP, Scholz R, Hesse V, Wittwer-Backofen U, Straube S, Olbertz D (2014) [New percentile values for the anthropometric dimensions of singleton neonates: analysis of perinatal survey data of 2007-2011 from all 16 states of Germany]. Zeitschrift für Geburtshilfe und Neonatologie 218:210-217.
3. Neuhauser H, Schienkiewitz A, Rosario AS, Dortschy R, Kurth B-M (2013) Referenzperzentile für anthropometrische Maßzahlen und Blutdruck aus der Studie zur Gesundheit von Kindern und Jugendlichen in Deutschland (KiGGS). Robert Koch-Institut. https://edoc.rki.de/handle/176904/3254
